# Advancements in n-Type Base Crystalline Silicon Solar Cells and Their Emergence in the Photovoltaic Industry

**DOI:** 10.1155/2013/470347

**Published:** 2013-12-29

**Authors:** Atteq ur Rehman, Soo Hong Lee

**Affiliations:** Green Strategic Energy Research Institute, Department of Electronic Engineering, Sejong University, 98 Gunja-dong, Gwangjin-gu, Seoul 143-747, Republic of Korea

## Abstract

The p-type crystalline silicon wafers have occupied most of the solar cell market today. However, modules made with n-type crystalline silicon wafers are actually the most efficient modules up to date. This is because the material properties offered by n-type crystalline silicon substrates are suitable for higher efficiencies. Properties such as the absence of boron-oxygen related defects and a greater tolerance to key metal impurities by n-type crystalline silicon substrates are major factors that underline the efficiency of n-type crystalline silicon wafer modules. The bi-facial design of n-type cells with good rear-side electronic and optical properties on an industrial scale can be shaped as well. Furthermore, the development in the industrialization of solar cell designs based on n-type crystalline silicon substrates also highlights its boost in the contributions to the photovoltaic industry. In this paper, a review of various solar cell structures that can be realized on n-type crystalline silicon substrates will be given. Moreover, the current standing of solar cell technology based on n-type substrates and its contribution in photovoltaic industry will also be discussed.

## 1. Introduction

An aluminium back surface field (Al-BSF) created by a co-fired screen-printed method and front side doped with phosphorus results in the currently dominating monocrystalline and multicrystalline silicon (mc-Si) solar cell structures. Although the photovoltaic industry is mainly occupied by p-type silicon substrates, it expects a growth in shares for n-type silicon substrates, as interest in using n-type silicon substrates has increased recently. The 2013 edition of the international technology road map for photovoltaics (ITRPV) also predicts a clear shift from p-type to n-type substrates in the market shares of monocrystalline silicon [1]. This expected shift in the solar cell module technology is because of certain significant advantages of n-type silicon over p-type silicon substrates for solar cell fabrication. The most important of these advantages offered by n-type silicon is the absence of boron oxygen-related, light-induced degradation (LID). It has already been reported that the boron oxygen pair formation causes degradation in carrier life time for c-Si solar cells based on p-type Czochralski (CZ) c-Si [2–4]. The absence of the boron in phosphorus-doped n-type substrates eliminates the boron oxygen defects even for the higher oxygen concentration. Furthermore, the n-type material exhibits enough strength against common impurities, such as interstitial Fe [5], which can capture the electrons much more effectively as it has a positive charge state. The minority carriers in n-type silicon are holes instead of electrons, as in the case of p-type silicon; therefore, it offers higher minority carrier diffusion lengths as compared to p-type c-Si substrates with similar impurity concentrations. Moreover, the use of a phosphorus-doped back surface field (BSF) with convenient surface passivation for such n-type cells, results in higher diffusion length and better rear internal reflection. The use of a boron-doped front emitter with rear side phosphorus-doped BSF on n-type substrates offers a bifacial type cell structure which can be fabricated on thinner wafers. The convenience of making such bifacial designed solar cells and modules using phosphorus-doped BSF, also generates opportunities to produce cells with higher efficiencies.

It is because of these benefits offered by n-type substrates that solar cells have already started to become attractive regarding the use of these substrates in solar cell module technology. Top companies like SunPower [6] and Sanyo [7] have already stepped in for manufacturing high efficiency commercialized solar cell modules using n-type c-Si substrates. The modules from both the companies are the highest efficiency solar cell modules available on the photovoltaic (PV) market. The Yingli Green Energy [8] has also adopted the use of n-type CZ c-Si substrates, coming up with the production of a high efficiency solar cell named PANDA, originally developed by the Energy Research Centre of the Netherlands (ECN) [9]. The interests in consideration of n-type substrates is growing as most of the major contributing companies and research organizations in the field of solar cells, including Bosch [10], Sunvia [11], and the Fraunhofer Institute of Solar Energy (ISE) [12], have already started to report advancements in cell processing from n-type substrates.

In this paper a brief review of the progression in the field of solar cells made from n-type base crystalline silicon solar cells will be given. Additionally, a detailed look at the industrially implemented n-type solar cells from SunPower, Sanyo and Yingli Green Energy, will be addressed.

## 2. Basic n-Type Cell Processing

The n-type materials for the solar cell fabrication process demands some additional care compared to solar cells fabricated on p-type substrates. In fact, the p-type substrates have some advantages in terms of the processing of solar cells, such as the convenience of phosphorus gettering, which assists improvement in cell efficiency, specifically for mc-Si wafers [13, 14]. The emitter formation in the case of n-type substrates has to be done via the boron diffusion process, which requires higher temperatures compared to the phosphorus diffusion for p-type cells, which makes the cell fabrication process more complex. Moreover, the process for two separate diffusion steps (emitter and BSF) renders it even more complicated and costly. During the boron diffusion process, another important issue is the formation of born rich layer (BRL) which is good for the gettering purpose but degrades the carrier lifetime in bulk [15]. Recently, a particularly effective method of removing BRL without the injection of gettering impurities has been developed [16, 17].

The passivation of boron-doped emitters was also a concern until recently, because the conventional method of passivating a P^+^ emitter by SiN_*x*_ is less effective [18]. The use of SiN_*x*_ results in the formation of positive fixed charges at SiN_*x*_/Si interface which leads to an increase in the surface recombination. Therefore, the passivation of boron-doped emitters is generally performed by thermal oxidation at the laboratory level, which is not considered suitable for low cost production. However, nowadays there are multiple methods that are feasible for effectively passivating the boron-doped emitter. One such method that recently received a lot of attention is the Al_2_O_3_ coating by ALD (atomic layer deposition) method, which introduces fixed negative charges at the Al_2_O_3_/Si interface [19–21]. A detailed review on the prospects for the use of Al_2_O_3_ as a passivation layer on crystalline silicon surfaces was given in [22]. Another effective passivation method that has been adopted [23] and implemented industrially [24] is the use of a low temperature acid-grown oxide, followed by a plasma enhanced chemical vapor deposition (PECVD) process that forms SiO_*x*_/SiN_*x*_ passivation stack on boron emitters.

When it comes to the metallization of the cell fabricated on n-type materials, the process becomes somewhat complex, since in order to form the n^+^ BSF at the rear of the cell, a phosphorus diffusion step is needed. Adding a phosphorus diffusion step at the rear side instead of using aluminum alloyed BSF (as in the case of p-type substrates) also requires a suitable diffusion mask at the front side of the cell. Apart from the complexity in cell processing, a better surface passivation with SiN_*x*_ at the rear of the cell can be acquired and good electrical contacts can be formed.

The resistivity variation through ingot due to segregation of dopants during crystal growth might be a concern for n-type cell processing. The growth of n-type ingots includes phosphorus dopant (having 0.35 segregation coefficients) leads to the higher resistivity variation through ingot, reducing its yield. However, boron-doped (having 0.7 segregation coefficients) p-type ingot has a lower resistivity variation, which is tolerable for the regular industrial solar cell process.

## 3. n-Type Solar Cell Structures 

**Figure 1:**
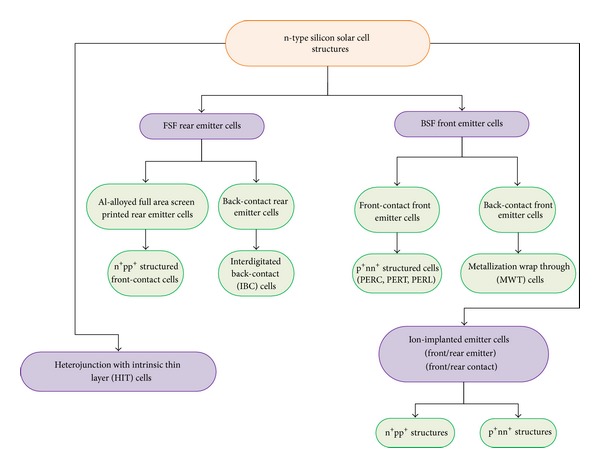
The flow chart of the possible cell structures using n-type silicon substrates.

There are a number of solar cell structures with higher efficiencies that have already been implemented successfully using n-type substrates.  Figure 1 illustrates these solar cell structures on n-type substrates briefly. The cell structures designed on n-type substrates will be discussed briefly in the preceding sections. These cell structures can be categorized according to the techniques used for cell processing and are described as follows:

1. front surface field (FSF) Al rear-emitter cells (n^+^np^+^ cells) can either have the contacts at the front or at the rear and normally has phosphorus diffused FSF;
2. back surface field (BSF) front-emitter cells (p^+^nn^+^ cells) can also have the contacts either on front or rear and are commonly boron-doped emitters with phosphorus-doped BSF;
3. ion implanted emitters cells have the emitter formed by ion implantation process and can be realized for both front and rear contact schemes on n^+^np^+^ and p^+^nn^+^ structures;
4. heterojunction with intrinsic thin-layer (HIT) cell structure.

## 4. Front Surface Field (FSF) Rear Emitter Cells 

### 4.1. Aluminum Alloyed Full Area Screen Printed Rear Emitter Cells

**Figure 2:**
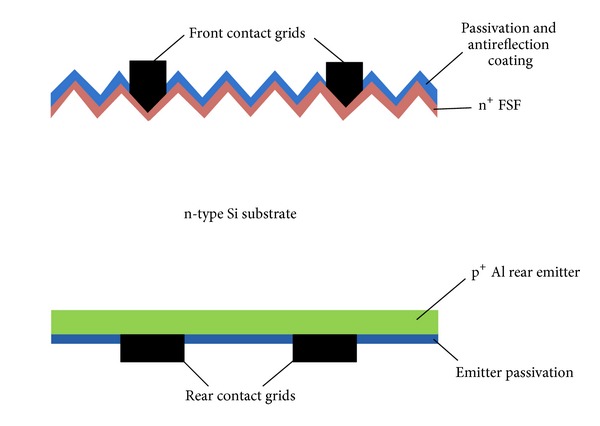
An n^+^np^+^ Al-rear emitter cells structure.

The easiest way to fabricate an n-type solar cell is by adopting the n^+^np^+^ structured rear emitter cell. Here, the FSF is made by phosphorus diffusion while the BSF (which act as p^+^ emitter of the cell) is formed using aluminium alloying by screen-printing on the rear side of the cell. As the contacts are formed on the rear side of the cell, the photo-generated carriers have to diffuse from the front to the rear of the cell through the bulk. Therefore, high quality wafers with excellent surface passivation on both sides is required to fabricate these cells. Glunz and coworkers at Fraunhofer ISE have presented such a rear-emitter solar cell. Even without using a rear-surface passivation, they were able to produce efficiencies of 19.3% [25] on an industrial scale and 19.8% [26] on a laboratory scale, on n-type float zone wafers. The rear recombination of such cell structures can be improved by adding a passivation layer to the rear emitter surface [27]. These passivating layers can account for the reduction of the emitter dark saturation current (*J*_oe_) with an increase in the internal reflectivity that can be useful for the improvement of cell efficiency. The investigation of improvements in cell efficiency by the use of passivation layers at the rear of the cell was done at Fraunhofer ISE, by considering two different passivating stacks at the rear emitter [28]. The passivation layers that are used to passivate the rear emitter of such cells were stacks of a-Si/SiO_*x*_ and Al_2_O_3_/SiO_*x*_. By adopting such passivation layers an improved cell efficiency of 20.1% for Al_2_O_3_/SiO_*x*_ and 19.5% for a-Si/SiO_*x*_ passivating stack layers was reported. The improvement in *V*_oc_ (25–30 mV for Al_2_O_3_ and 15–20 mV for a-Si/SiO_*x*_) in comparison to the non-passivated emitters was also observed. An n^+^np^+^ solar cell structure having a p^+^ emitter (with a-Si passivated) cell with an independent confirmed conversion efficiency of 19.7% on a CZ wafer was also successfully reported (Figure 2) [29].

### 4.2. Back-Contact Rear Emitter Cells

One of the most promising high efficiency modules on c-Si substrates can be formed with a solar cell that has hidden electrodes. The most effective way to hide these electrodes is to move the cell contacts to rear of the cell, due to which the absorption of light can be increased. This increase in absorption of light is because of the exclusion of the front side shading losses. As the front contacts of the cell can eventually reduce the cell efficiency by certain shading losses on the front surface of the cells [30]. Such a back contact solar cell requires higher carrier diffusion lengths as the carriers generated from the front surface have to transverse the entire wafer thickness in order to reach the rear emitter of the cell. The key design for such back-contact rear emitter cells are (i) locally defined rear contacts with lowered contact recombination losses, (ii) a grid free wafer surface which privileges the expansion of light trapping and passivation, and (iii) a rear side contacting scheme that enhances rear surface reflection and very low series resistance. The phosphorus-doped FSF for such back-contact rear emitter cells have a vital role in the improvement in cell efficiency, which reduces the lateral resistance losses significantly. This ability of reducing the lateral resistance losses was investigated at Fraunhofer ISE and reported with a best cell efficiency of 21.3% [31].

**Figure 3:**
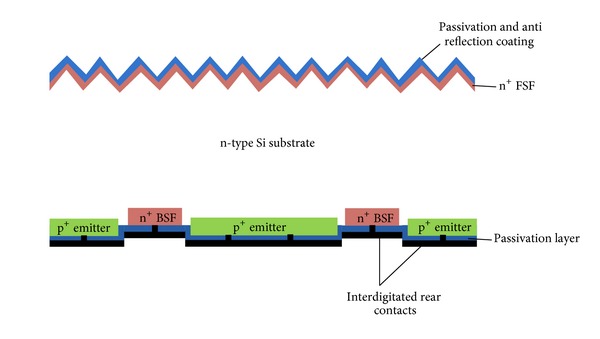
Schematic of back-junction back-contact n-type base c-Si solar cell. The rear contact is designed as an interdigitated back contact such as IBC cell from SunPower. This cross-section of IBC cell developed at ISFH shows an open front side n^+^-FSF, passivated and coated with antireflection coating (ARC).

The cell design in which the contacts that are moved to the structured p^+^ emitter formed at the rear surface can eventually result in the formation of a back-junction back-contact cells design, as the one shown in Figure 3. These cells can be categorized into those with an emitter formed from aluminium doping by screen printing or those with the emitters formed by boron diffusion process. SunPower is involved in producing such high efficiency interdigitated back contact solar cells commercially. The IBC solar cell adopted for industrial applications by SunPower will be discussed in detail in a later section about industrially implemented solar cells. Apart from IBC cells, the concept was adopted for research by various research groups and they reported their progress on such structures (Table 1) [33, 41–44]. The highest efficiency of over 24% has been achieved by an IBC cell from SunPower [34]. In these reports, different processing techniques were adopted for the formation of the rear emitter as well as the rear contacts for such rear emitter cells. The back-junction rear emitter cells having these Al-doped rear emitters formed by screen printing the aluminium paste by either structuring the emitter before [41] or after the application of the aluminium paste [42], resulting in 19.1% and 19.0% efficient cells on CZ wafers, respectively. This process was successfully done at the Institute for Solar-energy Research Hamelin (ISFH) [45]. As it has been already discussed that the passivation of such a back-contact cell is very important, as the improvement in cell efficiency can be accomplished by high quality passivation, advanced passivation schemes for such cells are currently under investigation by several groups including ECN [43].

Woehl et al. also demonstrated an industrially feasible process by metallizing the rear emitter within the screen printing step [44]. In the fabrication process they adopted for their back contact rear emitter cell, they printed the rear surface by using the screen printing resist and both (Ag and Al) metallization pastes at the same time. By bringing in the metallization altogether with the screen printing step, it would not only be cost effective but it could be very advantageous for industrial applications as well. The 20% efficient cells were reported by the same group for adopting the process of metallizing the cell within the screen printing step using an n-type float-zone (FZ) silicon wafer [33].

## 5. Back Surface Field (BSF) Front Emitter Cells

### 5.1. Front Emitter Front Contact Cells

The solar cell on n-type substrate can also be realized by just converting the conventional p-type solar cell to a p^+^nn^+^ structure. The p^+^ emitter at the front of these cells is generally formed by boron-diffusion while the n^+^-BSF at the rear is set up by phosphorus diffusion. The high efficiencies that can be obtained by n-type substrates in comparison to typical Al-alloyed BSF p-type cells are due to certain factors. The p^+^nn^+^ structures have a low emitter recombination current, superior diffusion lengths, and much better internal rear reflection than Al-BSF of p-type cells. The rear fully diffused with phosphorus provides an opportunity of bifacial design, which enables the possibility of light collection from both sides. Solar cells having efficiencies exceeding 20% using p^+^ boron-doped emitters are already developed in laboratory settings with a different cell structure. At the University of New South Wales (UNSW), the passivated emitter rear totally diffused (PERT) solar cell having a p^+^nn^+^ type solar cell structure was successfully implemented using an n-type substrate [36]. The PERT type cells fabricated at UNSW have conversion efficiencies of 21.9% and 21.1% on FZ and CZ n-type substrates, respectively. Fraunhofer ISE was also involved in fabricating a high efficiency solar cell on n-type substrates by adopting the passivated emitter rear locally diffused (PERL) structure and reported efficiencies of 23.4% [46] and 23.9% [35] with Al_2_O_3_ passivation at the emitters of the cells.

**Figure 4:**
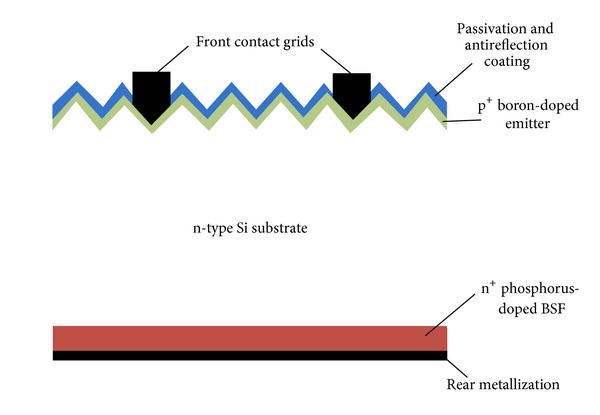
Cross-section of p^+^nn^+^ structured solar cell. The emitters of such solar cells are usually formed by boron diffusion process. The n^+^ phosphorus doped (BSF) at the rear provides an opportunity for a bifacial designed solar cell.

The passivation and contacting of the boron-doped n-type emitter surface is much more challenging as compared to the p-type surface. The regular passivation layer SiN_*x*_ has failed to accomplish good results [47] as well as the thermal oxidation process [47, 48] for boron-doped emitters. In the current scenario, a strong candidate to passivate boron-doped emitters effectively is Al_2_O_3_, as it is capable of introducing fixed negative charges at the emitter surface. It has been reported by Hoex et al. that by adopting the deposition technique of Al_2_O_3_ by ALD, can reduce the emitter dark saturation current (*J*_oe_) efficiently, and a lower *J*_oe_ value of about 10 fA/cm^2^ was reported by them in [20]. The Al_2_O_3_/SiN_*x*_ layers stack passivation over boron emitter was also used and it resulted in lowering the *J*_oe_ value to 25 fA/cm^2^ with *V*_oc_ higher than 700 mV and an efficiency of 20.4% [49]. Apart from using Al_2_O_3_, the use of an ultrathin layer of SiO_2_ which was grown in nitric acid followed by SiN_*x*_ deposition to passivate the boron-doped emitter has also been developed at ECN [22]. Regardless of the available techniques, the industrial implementation feasibility of these passivation methods is a big concern, where cost as well as throughput has importance. The manufacturing of p^+^nn^+^ bifacial cell with adaptable industrial techniques was embraced by many institutes recently (Figure 4) [23, 24, 35, 50, 51].

### 5.2. Front Emitter Back-Contact Cells

It has already been realized that by moving the contacts to the rear of the cell, it can eventually increase the efficiency of the cell due to the reduction of the shading losses at the front. The metallization wrap-through (MWT) and emitter wrap-through (EWT) are the cells based on the back contact front emitter technology. The MWT technology has been adopted to be manufactured on n-type substrates recently. These MWT cell structures are composed of metal grids on the front surface with the presence of linkage pads for both terminals at the rear surface. In these cells, the front shading losses are not fully eliminated; however, these losses are significantly reduced by avoiding the interconnect pads and solder strips. The connection between front metal grids with the corresponding pads can be formed over the edge of the wafer or through holes [52, 53] or slots [54] shaped in the substrates.

It poses certain advantages over other back contact technology such as IBC cells from SunPower. For example, apart from the complexity of the cell processing, IBC cells also require very high quality substrates as well as surface passivation and accuracy for aligning the metal contacts at the rear. The MWT cell concept has the advantage of using a cell process similar to the conventional cell processing and simpler rear side contact patterns. Moreover the structure having an emitter at the front can compromise using the lower quality materials.

**Figure 5:**
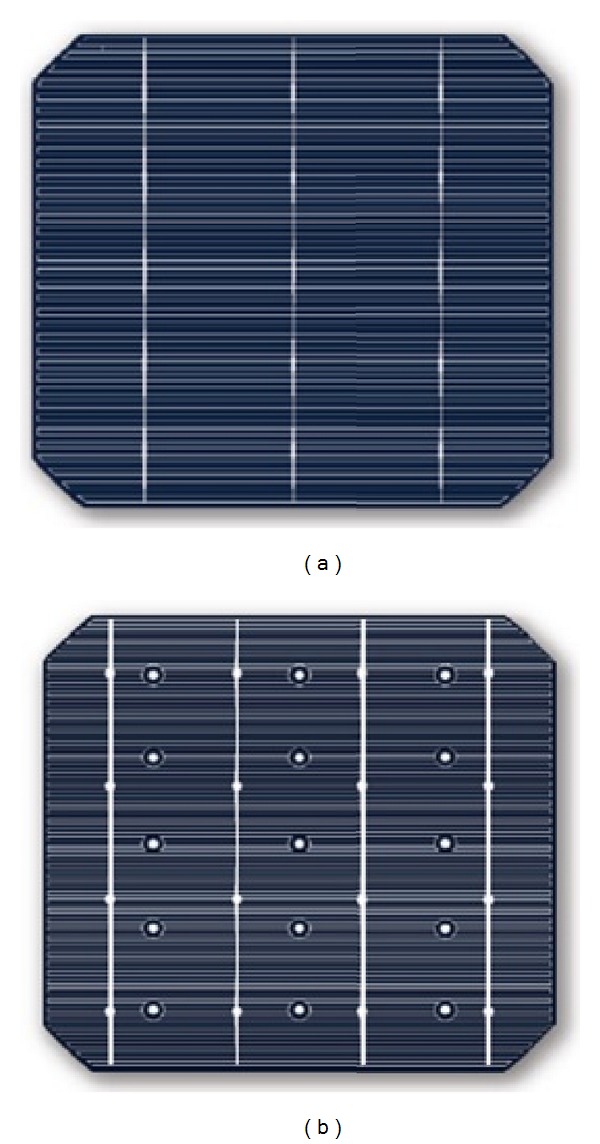
Image of n-type MWT silicon solar cells with a H-pattern-based unit cell design. Front side (a) and rear side (b) [38].

ECN has been involved in the production of n-type MWT cells and reported an efficiency of up to 19.7% with low cost industrial techniques on CZ wafers [38]. There have been reports on the MWT n-type technology for industrial applications by various authors [55, 56]. The bifacial modules with superior performance can also be manufactured using MWT back-contact technology, which can offer modules having an efficiency close to 20% or higher. A bifacial industrial type MWT solar cell with 0.35% absolute gain was successfully fabricated at Yingli Green Energy recently (Figure 5) [57].

## 6. Ion Implanted Emitter Cells 

**Figure 6:**
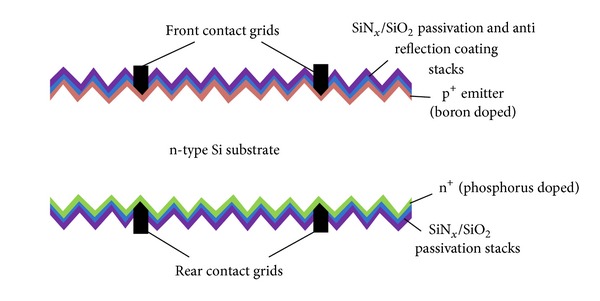
Structure of boron front emitter (p^+^nn^+^) type cell. Such solar cell was fabricated by research team at Sunvia solar, where an ion-implantation technique was used for implanting the boron-doped emitter and phosphorus-doped BSF [58].

Ion implantation is a developed technology used in the CMOS fabrication for decades and could be a promising approach for solar cell fabrication. The accurate control with patterned single side process of ion implantation can be used to fabricate state of the art high efficiency structures such as interdigitated back contact (IBC) and selective emitter solar cells. The generation of n^+^ or p^+^ doped emitters can be done by means of ion implantation. The formation of the emitters using an ion implantation technique requires quite low ion doses for a short time in comparison to other doping techniques. The ion implantation can be useful for industrial applications as it offers certain benefits which are demonstrated well in [62]. The most important one is the formation of an accurate doping profile with a uniformly doped emitter. The central concern of ion implantation is the elimination of the contaminants introduced by the implantation mechanism, since the high efficiency solar cells need a well-passivated fault free surface. The annealing step after the formation of doped emitter using implantation is essential in order to remove this damage introduced by the implantation. The annealing step is critical, because it can be handy for obtaining the low emitter dark saturation currents (*J*_oe_). If the ion implanted samples are annealed under the condition with a suitable high temperature, then the emitter dark saturation currents (*J*_oe_) can be lowered down quite effectively. The Benick et al. research team at Fraunhofer ISE was able to fabricate such high efficiency cells with lower dark saturation currents of up to 24 fA/cm^2^ for boron-doped emitters [63, 64]. Another very good feature of using ion implantation for doping purposes is in situ masking of implantation which allows the formation of the locally defined doping profiles quite easily (Figure 6).

The high through put of up to 1000 wafers (156 mm pseudo-square) per hour [65] can be achieved by using the ion-implantation process, which can provide an opportunity to obtain grid parity. The fabrication of advanced cell structures (i.e., selective emitters) can be simplified by using the ion implantation process. The comfort and ease of cell processing with benefits such as reductions in process steps, improved blue response, edge isolation elimination, and a uniformly doped emitter can be formed by this ion implantation process [62]. Sunvia Inc. working in collaboration with Varian Semiconductor Equipment Associates (VSEA) [66] and the Georgia Institute of Technology has pioneered the ion-implantation technology. They were able to achieve a 19.1% efficiency for selective emitter solar cells based on n-type silicon substrate [67]. Higher efficiencies greater than 19% have also been reported by Rohatgi and Meier [68]. The local doping for the IBC cells can be performed with ease and comfort by using an ion-implantation technique, Bateman et al. at Varian fabricated and reported a 20% efficient IBC cell with high quality implanted boron emitters by using such an ion implantation technique [40].

## 7. Commercial n-Type Silicon Solar Cell Technology

During the last several years, there have been only two companies (SunPower and Sanyo) commercializing the solar cell technology based on n-type substrates successfully. Sanyo is producing the modules based on HIT solar cells, while SunPower is manufacturing the modules using IBC solar cells. Recently, Yingli Green Energy has also stepped in for the commercialization of high efficiency cells named “PANDA” solar cells based on n-type materials. In this section, a review of the n-type Si solar cells produced in mass production by Sanyo, SunPower, and Yingli solar will be given.

### 7.1. HIT n-Type Si Substrates Solar Cells

**Figure 7:**
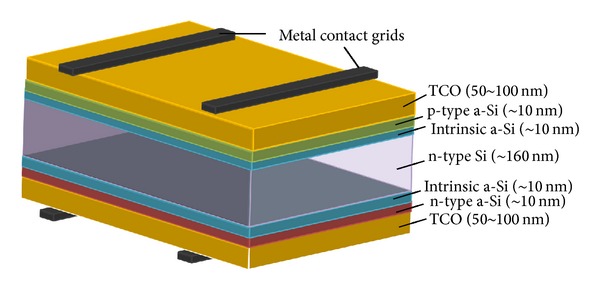
HIT solar cell structure.

The HIT cell based on n-type Si substrates was proposed by Sanyo Electronic Corporation and mass production of this cell was launched commercially in 1997. The HIT cell structure is composed of an intrinsic amorphous-Si layer, a doped amorphous-Si layer, followed by a transparent conductive oxide (TCO) layer deposited on both sides of an n-type Si wafer. The silver grid electrodes are also formed on both sides of the cell. The structure formed by this process for HIT solar cell is a symmetrical structure, which can also be realized as a bifacial solar cell that can lead to higher harvestings of solar power over the system life span [69]. The schematic of HIT solar cell is shown in Figure 7 [70].

**Figure 8:**
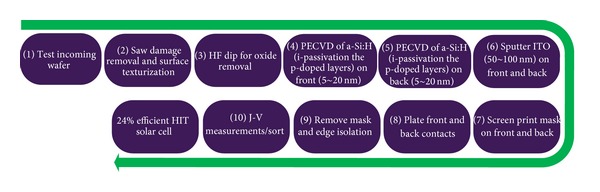
Model fabrication process flow for bifacial technology HIT solar cell [59].

The fabrication process for HIT solar cells is different than standard p^+^nn^+^ cell structure based on n-type wafers (which uses high temperature diffusion processes for cell development). Lower temperatures (below 200°C) [71] with very simple sequenced fabrication process as compared to the conventional crystalline Si solar cells are required to fabricate the n-type HIT solar cell structures. The model process flow for such bifacial solar cells is shown in Figure 8, which is adopted from [59].

The efficiency of HIT solar cell by Sanyo reached a level of 23.7% for a size of (<100.4 cm^2^) substrate [39]. They developed special technologies by using the thinner wafers, by achieving a high conversion efficiency of 22.8% using only a 98-*μ*m-thick substrate [72]. The research team in Sanyo also commercialized the HIT solar cell at the mass-production stage and have reached the production of 240-W model technology with a cell conversion efficiency of 21.6% and module conversion efficiency of 19.0% [73].

### 7.2. IBC n-Type Si Substrates Solar Cells

**Figure 9:**
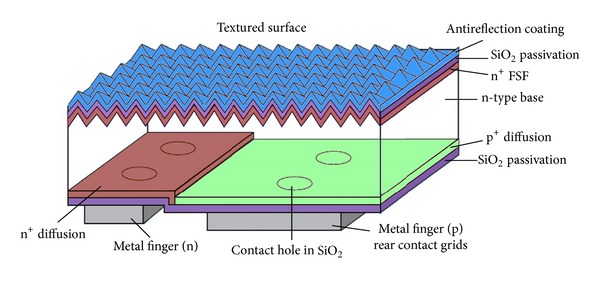
Schematic sketch of the IBC solar cell from SunPower [60].

The A-300 solar cell commercialized by SunPower was originally developed at Stanford University. The IBC cell based on the rear contact technology from SunPower is shown in Figure 9 [60]. As it has already been understood that moving the contacts to the rear of the cell can eventually increase the cell efficiency, which is the main feature of IBC cell design. The cell having no contacts at the front exposes all the front area of the cell and allows the sunlight to generate carriers with no shading losses. These cells are comprised of a back contact rear emitter design, thus nearly all the generated carriers at the front have to move through the bulk to the rear of the cell. Therefore, the minority carrier diffusion lengths of the bulk material have to be high and surface recombination velocity at the front has to be the minimal. Regardless of the higher efficiencies offered by these cells they also demand high quality materials and technology. Better surface passivation especially at the front surface is greatly required in order to reduce the front surface recombination. For this purpose, an excellent SiO_2_ passivation with good dielectric properties [74] can be used as a passivating material at the front surface. The formation of phosphorus-doped n^+^-FSF is usually done by POCl_3_ [75] diffusion. SiN_*x*_/SiO_2_ stacks are used as a passivation layer which can enhance the efficiency because of having high quality surface passivation and higher transparency to the sunlight with better antireflection properties. The rear side passivation of boron-doped emitter is done by SiO_2_ by forming a thick layer of Si/SiO_2_ on back which can provide higher reflectivity at the rear.

The A-300 IBC solar cell was first manufactured and reported by SunPower in 2004 on a large area (149 cm^2^) having a maximum cell efficiency of 21% [60]. In 2007, De Ceuster et al. team at SunPower introduced a new generation of A-300 IBC cells on a large volume production having an average efficiency of 22.4% [76]. These IBC cells hold the records of having highest efficiency silicon solar cell in a mass production so far. In the same article a high module efficiency of 20.1% using IBC solar cells was also reported. In 2010, a research team at SunPower reported a new record efficiency of 24.2% at R and D level [34].

### 7.3. PANDA n-Type Si Substrates Solar Cell

**Figure 10:**
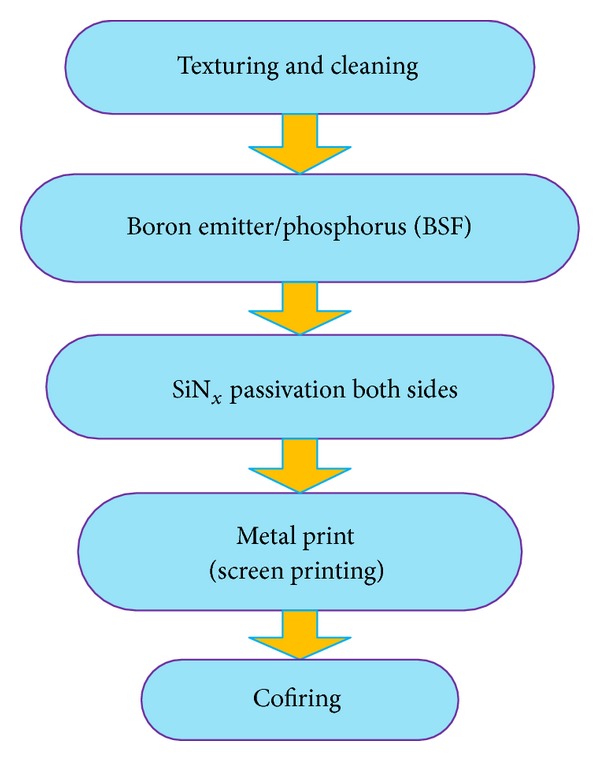
Flow chart of PANDA solar cell manufacturing [50].

The PANDA solar cell technology has been moved to pilot production by Yingli Green Energy recently and is a joint project between Yingli Green Energy, ECN and Amtech Systems. The PANDA solar cell is actually ECN's, a laboratory scale n-type Si solar cell with low cost features including a boron diffused front emitter, phosphorus BSF as well as front and rear screen printed electrodes [77]. The schematic and process flow chart of ECN's, PANDA solar cell is shown in Figure 8. The cell design is comprised of transparent phosphorus BSF with an open rear contact, making it a bifacial cell structure, which increases the possibility of light injection and enhances the power generating capacity. The PANDA solar cell technology has certain advantages compared to the established n-type Si solar cell technologies: it is easy to process, requires lower cost, and is compatible with the current p-type Si cell processing lines. It has the ability to produce higher power due to its bifacial design and its symmetrical structure also makes it suitable for processing on thin wafers. A comparison between an n-type Si PANDA solar cell module and conventional p-type Si module has been made in [61], which clearly demonstrates the superiority of the PANDA solar cell module having zero LID, high temperature performance and better module efficiencies even at lower irradiance (Figure 10).

**Figure 11:**
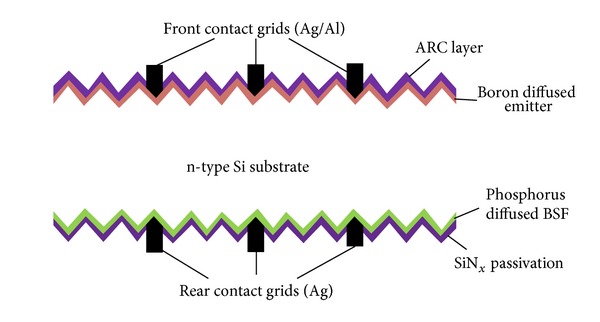
Schematic of Yingli's PANDA solar cell [61].

The Yingli Green Energy made efforts to commercialize the high efficiency PANDA solar cell technology with a lower manufacturing cost. By taking certain improvement steps they were able to produce the PANDA solar cell on commercial production lines with 19% stable average efficiency using semi square 6-inch n-type CZ wafers [37]. PANDA 325 modules were commercially available in the middle of 2010 and were certified to the international photovoltaic standards (Figure 11).

ECN is currently working on the development of highly efficient, low price module concepts that can easily be adapted to the industrial environment. The n-Pasha (passivated all sides H-pattern) cell concept on CZ n-type wafers was introduced by ECN in 2010. By 2012, an efficiency rate of 20% at reduced costs per watt-peak (*W*_*p*_) was achieved by the research team at ECN [78]. More recently, in a report from the research group at ECN, a highly efficient n-Pasha cell with reduced costs has been fabricated. The reduction in silver consumption for the front and rear metallization was put into practice in order to develop solar cell modules that had 20% efficiency and lower costs [79].

## 8. Conclusions 

The article reviews recent progress made in solar cell technology based on n-type crystalline silicon substrates. It can be clearly inferred that n-type material has the potential to compete with the existing technology based on p-type substrates. The n-type substrates offer higher efficiencies due to material properties that can boost solar cell performance. The most compelling reasons to prefer n-type silicon over p-type are (1) the avoidance of boron-oxygen related LID and (2) greater tolerance against common metal impurities. Currently, the most important concern is the development of low cost n-type cell processing technology for industrial applications, in order to reduce the cost per watt-peak (*W*_*p*_). In this regard several institutes are presently engaged in determining such cost effective n-type cell technologies. The most progressive of these institutes being PANDA technology developed by ECN and Yingli. ECN and Yingli solar have also been successful in recently developing the bifacial n-Pasha cell technology. Therefore, the higher efficiencies offered by the use of technology based on n-type materials, along with the new exciting improvements and results, clearly indicate that n-type solar cells will rise as a major contributor to market share in coming years.

## Figures and Tables

**Table 1:** Best results for various n-type high efficiency cell structures.

Type	Structure	Metallization	*V*_oc_ (mV)	Efficiency (%)	References
Front surface field (FSF) rear emitter cells	n^+^np^+^ PERT (rear emitter cell)	Plated Ag metallization	702	22.7	[32]
	n^+^np^+^ (Al rear emitter)	Front evaporated TiPdAg, Rear full area evaporated	649	20.1	[28]
	n^+^np^+^ (back contacts)	Screen printed	647	20.0	[33]

Back surface field (BSF) front emitter cells	IBC		721	24.2	[34]
	PERL (p^+^nn^+^)	Evaporated front grid, rear full area evaporated	705	23.9	[35]
	PERT (p^+^nn^+^)	Evaporated front grid + plating, rear full area evaporated	695	21.9	[36]
	p^+^nn^+^ (PANDA cell)	Stencil printed	649	20.0	[37]
	MWT (p^+^nn^+^) (back contacts)	Screen printed	644	19.7	[38]

Heterojunction with intrinsic thin layer (HIT) solar cell	HIT cell	Screen printed	745	23.7	[39]

Ion implanted emitter cells	IBC	Evaporated Al/Ti/Pd/Ag	650.1	20.0	[40]

## References

[1] International Technology Roadmap for Photovoltaic (ITRPV) http://www.itrpv.net/.

[2] Schmidt J, Bothe K (2004). Structure and transformation of the metastable boron- and oxygen-related defect center in crystalline silicon. *Physical Review B*.

[3] Glunz SW, Rein S, Lee JY, Warta W (2001). Minority carrier lifetime degradation in boron-doped Czochralski silicon. *Journal of Applied Physics*.

[4] Schmidt J, Aberle AG, Hezel R Investigation of carrier lifetime instabilities in Cz-grown silicon.

[5] Macdonald D, Geerligs LJ (2004). Recombination activity of interstitial iron and other transition metal point defects in p- and n-type crystalline silicon. *Applied Physics Letters*.

[6] SUNPOWER http://us.sunpowercorp.com/.

[7] Sanyo Solar http://panasonic.net/energy/solar/.

[8] Yingli Solar http://www.yinglisolar.com/.

[9] Energy Research Centre of Netharland (ECN) http://www.ecn.nl/home/.

[10] Bosch Solar Energy http://www.bosch-solarenergy.com/en/bosch_se_online/landing_page/landing_page_1.html.

[11] Sunvia Solar http://www.suniva.com/.

[12] http://www.ise.fraunhofer.de/en.

[13] Lotfi D, Hatem E (2012). Phosphorus diffusion gettering process of multicrystalline silicon using a sacrificial porous silicon layer. *Nanoscale Research Letters*.

[14] Phang SP, MacDonald D (2011). Direct comparison of boron, phosphorus, and aluminum gettering of iron in crystalline silicon. *Journal of Applied Physics*.

[15] Kessler MA, Ohrdes T, Wolpensinger B, Harder N (2010). Charge carrier lifetime degradation in Cz silicon through the formation of a boron-rich layer during BBr3 diffusion processes. *Semiconductor Science and Technology*.

[16] Ryu K, Upadhyaya A, Song HJ, Choi CJ, Rohatgi A, Ok YW (2012). Chemical etching of boron-rich layer and its impact on high efficiency n-type silicon solar cells. *Applied Physics Letters*.

[17] Phang SP, Liang W, Wolpensinger B, Kessler MA, Macdonald D (2013). Tradeoffs between impurity gettering, bulk degradation, and surface passivation of Boron-Rich layers on silicon solar cells. *IEEE Journal of Photovoltaics*.

[18] Chen FW, Li TA, Cotter JE (2006). Passivation of boron emitters on n-type silicon by plasma-enhanced chemical vapor deposited silicon nitride. *Applied Physics Letters*.

[19] Hoex B, Gielis J, Van de Sanden MCM, Kessels WM (2008). On the c–Si surface passivation mechanism by the negative-charge-dielectric Al_2_O_3_. *Journal of Applied Physics*.

[20] Hoex B, Schmidt J, Bock R, Altermatt PP, Van De Sanden MCM, Kessels WMM (2007). Excellent passivation of highly doped p-type Si surfaces by the negative-charge-dielectric Al_2_O_3_. *Applied Physics Letters*.

[21] Hoex B, Heil SBS, Langereis E, Van De Banden MCM, Kessels WMM (2006). Ultralow surface recombination of c–Si substrates passivated by plasma-assisted atomic layer deposited Al_2_O_3_. *Applied Physics Letters*.

[22] Dingemans G, Kessels E (2012). Status and prospects of Al_2_O_3_-based surface passivation schemes for silicon solar cells. *Journal of Vacuum Science & Technology A*.

[23] Mihailetchi VD, Komatsu Y, Geerligs LJ (2008). Nitric acid pretreatment for the passivation of boron emitters for n-type base silicon solar cells. *Applied Physics Letters*.

[24] Mihailetchi VD, Jourdan J, Edler A In Screen printed n-type silicon solar cells for industrial application.

[25] Schmiga C, Rauer M, Rüdiger M In Aluminium-doped p^+^ silicon for rear emitters and back surface fields: results and potentials of industrial n-and p-type solar cells.

[26] Schmiga C, Hörteis M, Rauer M In Large-area n-type silicon solar cells with printed contacts and aluminium-alloyed rear emitter.

[27] Rauer M, Schmiga C, Hermle M, Glunz SW In Passivation of screen-printed aluminium-alloyed emitters for back junction n-type silicon solar cells.

[28] Schmiga C, Hermle M, Glunz SW In Towards 20% efficient n-type silicon solar cells with screen-printed aluminium-alloyed rear emitter.

[29] Bock R, Schmidt J, Brendel R (2008). N-type silicon solar cells with surface-passivated screen-printed aluminium-alloyed rear emitter. *Physica Status Solidi*.

[30] Swanson RM Developments in silicon solar cells.

[31] Granek F, Hermle M, Huljić DM, Schultz-Wittmann O, Glunz SW (2009). Enhanced lateral current transport via the front N+ diffused layer of N-type high-efficiency back-junction back-contact silicon solar cells. *Progress in Photovoltaics*.

[32] Zhao J, Wang A High efficiency rear emitter pert cells on CZ and FZ n-type silicon substrates.

[33] Woehl R, Keding R, Rudiger M In 20% Efficient screen-printed and aluminum-alloyed back-contact back-junction cells and interconnection scheme of point-shaped metalized cells.

[34] Cousins P, Smith D, Luan H-C Generation 3: improved performance at lower cost.

[35] Glunz S, Benick J, Biro D n-Type silicon-enabling efficiencies >20% in industrial production.

[36] Zhao J, Wang A, Altermatt PP, Green MA, Rakotoniaina JP, Breitenstein O High efficiency pert cells on n-type silicon substrates.

[37] Geerligs L, Romijn IG, Burgers A In Progress in low-cost n-type silicon solar cell technology.

[38] Guillevin N, Heurtault BJB, Geerligs LJ, Weeber AW (2011). Development towards 20% efficient Si MWT solar cells for low-cost industrial production. *Energy Procedia*.

[39] Kinoshita T, Fujishima D, Yano A In The approaches for high efficiency HIT solar cell with very thin (<100 *μ*m) silicon wafer over 23%.

[40] Bateman N, Sullivan P, Reichel C, Benick J, Hermle M High quality ion implanted boron emitters in an interdigitated back contact solar cell with 20% efficiency.

[41] Gong C, Van Kerschaver E, Robbelein J (2010). Screen-printed aluminum-alloyed P+ emitter on high-efficiency N-type interdigitated back-contact silicon solar cells. *IEEE Electron Device Letters*.

[42] Bock R, Mau S, Schmidt J, Brendel R (2010). Back-junction back-contact n -type silicon solar cells with screen-printed aluminum-alloyed emitter. *Applied Physics Letters*.

[43] Castaño F, Morecroft D, Cascant M In Industrially feasible >19% efficiency IBC cells for pilot line processing.

[44] Woehl R, Krause J, Granek F, Biro D (2011). 19.7% efficient all-screen-printed back-contact back-junction silicon solar cell with aluminum-alloyed emitter. *IEEE Electron Device Letters*.

[45] http://www.isfh.de/index.php?dm=1&&_l=1.

[46] Benick J, Hoex B, Dingemans G In High-efficiency n-type silicon solar cells with front side boron emitter.

[47] Altermatt P, Plagwitz H, Bock R In The surface recombination velocity at boron-doped emitters: comparison between various passivation techniques.

[48] Zhao J, Schmidt J, Wang A, Zhang G, Richards BS, Green MA Performance instability in n-type pert silicon solar cells.

[49] Richter A, Henneck S, Benick J, örteis M H, Hermle M, Glunz S In Firing stable Al_2_O_3_/SiNx layer stack passivation for the front side boron emitter of n-type silicon solar cells.

[50] Burgers A, Geerligs L, Carr A In 19.5% efficient n-type si solar cells made in production.

[51] Burgers A, Naber R, Carr A In 19% efficient n-type Si solar cells made in pilot production.

[52] Lillington D, Kukulka J, Bunyan S, Garlick G, Sater B In Development of 8 cm × 8 cm silicon gridded back solar cell for space station.

[53] Lillington DR, Kukulka JR, Mason AV, Sater BL, Sanchez J Optimization of silicon 8 cm x 8 cm wrapthrough space station cells for “on orbit” operation.

[54] Cavicchi B, Mardesich N, Bunyan S In Large area wraparound cell development.

[55] Guillevin N, Heurtault B, van Aken B (2012). High efficiency n-type metal wrap through cells and modules. *Energy Procedia*.

[56] Guillevin N, Geerligs L, Naber R, Eerenstein W, Weeber A In High efficiency n-type metal wrap through Si solar cells for low-cost industrial production.

[57] Zhao W, Wang J, Shen Y In 0.35% Absolute efficiency gain of bifacial N-type Si Solar cells by industrial metal wrap through technology.

[58] Meier DL, Chandrasekaran V, Davis HP (2011). n-type, ion-implanted silicon solar cells and modules. *IEEE Journal of Photovoltaics*.

[59] Goodrich A, Hacke P, Wang Q (2013). A wafer-based monocrystalline silicon photovoltaics road map: utilizing known technology improvement opportunities for further reductions in manufacturing costs. *Solar Energy Materials and Solar Cells*.

[60] Mulligan WP, Rose DH, Cudzinovic MJ In Manufacture of solar cells with 21% efficiency.

[61] Song D, Xiong J, Hu Z In Progress in n-type Si solar cell and module technology for high efficiency and low cost.

[62] Low R, Gupta A, Bateman N High efficiency selective emitter enabled through patterned ion implantation.

[63] Benick J, Richter A, Li T-TA Effect of a post-deposition anneal ON Al_2_O_3_/Si interface properties.

[64] Benick J, Bateman N, Hermle M In Very low emitter saturation current densities on ion implanted boron emitters.

[65] Gupta A, Low R, Bateman N In High efficiency selective emitter cells using in-situ patterned ion implantation.

[66] http://www.vsea.com/.

[67] Rohatgi A, Meier DL, McPherson B High-throughput ion-implantation for low-cost high-efficiency silicon solar cells.

[68] Rohatgi A, Meier D (2010). Developing novel low-cost, high-throughput processing techniques for 20%-efficient monocrystalline silicon solar cells. *Photovoltaics International*.

[69] Taguchi M, Kawamoto K, Tsuge S (2000). HIT cells—high-efficiency crystalline Si cells with novel structure. *Progress in Photovoltaics*.

[70] Mishima T, Taguchi M, Sakata H, Maruyama E (2011). Development status of high-efficiency HIT solar cells. *Solar Energy Materials and Solar Cells*.

[71] Maruyama E, Terakawa A, Taguchi M Sanyo’s challenges to the development of high-efficiency HIT solar cells and the expansion of HIT business.

[72] Taguchi M, Tsunomura Y, Inoue H In High efficiency HIT solar cell on thin (<100 *μ*m) silicon wafer.

[73] Maki K, Fujishima D, Inoue H In High-efficiency HIT solar cells with a very thin structure enabling a high *V*
_oc_.

[74] Yablonovitch E, Gmitter T, Swanson RM, Kwark YH (1985). A 720 mV open circuit voltage SiOx:c–Si:SiOx double heterostructure solar cell. *Applied Physics Letters*.

[75] King RR, Sinton RA, Swanson RM (1990). Studies of diffused phosphorus emitters: saturation current, surface recombination velocity, and quantum efficiency. *IEEE Transactions on Electron Devices*.

[76] De Ceuster D, Cousins P, Rose D, Vicente D, Tipones P, Mulligan W In Low Cost, high volume production of >19% efficiency silicon solar cells.

[77] Weeber A, Naber R, Guillevin N In Status of n-type solar cells for low-cost industrial production.

[78] Romijn IG, Aken BBvan, Anker J In Industrial implementation of efficiency improvements in n-type solar cells and modules.

[79] Romijn I, Anker J, Burgers A In Industrial n-type solar cells with >20% cell efficiency.

